# Alveolar soft part sarcoma of the tongue in a 3-year-old boy: a case report

**DOI:** 10.1186/1752-1947-4-130

**Published:** 2010-05-08

**Authors:** George Noussios, Pantelis Chouridis, Ioannis Petropoulos, Kostas Karagiannidis, George Kontzoglou

**Affiliations:** 1 ENT Department, (Street Konstantinoupoleos 49) Hippokratio General Hospital, Thessaloniki, Greece; 2 Department of Physical Education (Serres) of 'Aristotelian' University, (Agios Ioannis-Serres), Thessaloniki, Greece

## Abstract

**Introduction:**

Alveolar soft tissue sarcoma of the tongue is a very rare and aggressive tumor which occurs in infancy with a relatively controversial histogenesis. It may mimic benign vascular neoplasms and may metastasize to the lungs, brain or bones.

**Case presentation:**

We present the case of a three-year-old Caucasian boy who was admitted to our department with a history of dysphagia and two episodes of oral bleeding which had lasted for a period of six months. A thorough histological examination together with imaging techniques form the basis of a reliable diagnosis.

**Conclusion:**

Alveolar soft tissue sarcoma of the tongue is a rare tumor which occurs in infancy and which is often misdiagnosed pre-operatively. It should therefore be included in the differential diagnosis of oral pediatric lesions.

## Introduction

Alveolar soft part sarcoma of the tongue (ASPS) is a rare and aggressive malignancy which comprises 0.4% to 1.0% of all soft tissue sarcomas. It accounts for up to 5% of all pediatric soft tissue sarcomas, apart from rhabdomyosarcomas [1]. In infants and children, the most common site of origin is the head and neck region, especially the orbit and tongue. It occurs extremely rarely in children under 5 years of age: only a dozen such cases have been reported in the last 50 years. We present a case of ASPS occurring in the dorsum of the tongue.

## Case report

**Figure 1:**
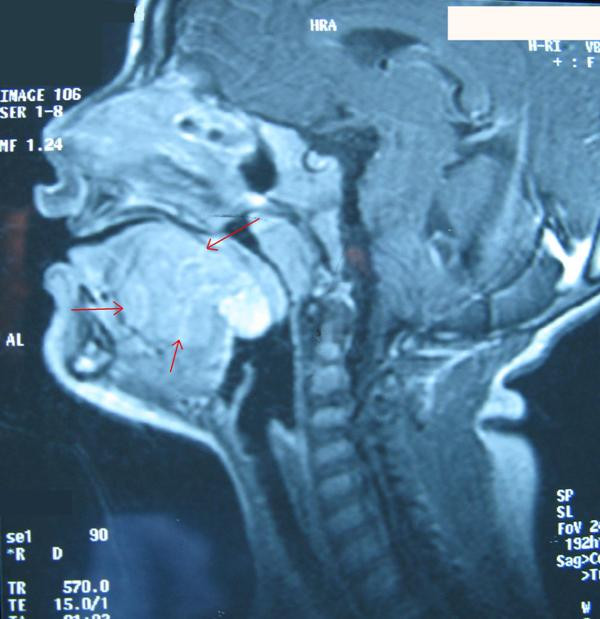
**Baccillary view of the mass (red arrow)**.

**Figure 2:**
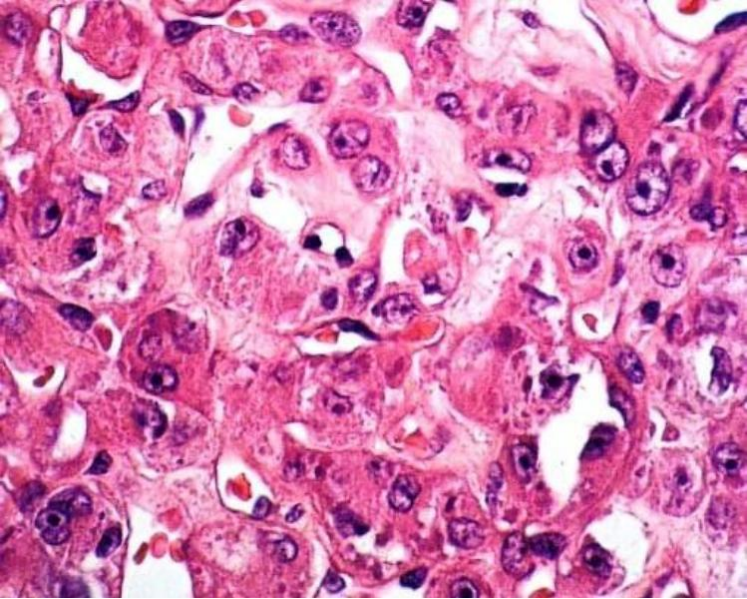
**Transversal view of the tumor (red arrow)**.

A 3-year-old Caucasian boy originating from Northern Greece was admitted to our department in Hippokratio General Hospital of Thessaloniki with a history of dysphagia and repeated episodes of oral bleeding which had lasted for a period of six months. A physical examination revealed a reddish-blue, soft, painful and immobilized mass in the mid-dorsum area of the tongue extending to its base. There was no cervical lymph node enlargement. Computed tomography (CT) and magnetic resonance imaging (MRI) demonstrated a 3.3×1.9×2.0 cm mass with new vessel generation. It was obstructing the entrance to his pharynx and was initially thought to be a hemangioma (Figures 1 and 2). A biopsy was performed under general anesthesia and histological examination showed a hemangioma of the tongue. He was treated conservatively with corticosteroids and interferon (IFN) 2A. A biopsy specimen was also sent to a hospital laboratory which specialized in vascular anomalies. Microscopic examination also showed a benign capillary hemangioma.

Despite the treatment, the mass had accreted excessively and six months later it was causing partial airway obstruction. Our patient was put in contact with another specialized center in pediatric tumors and he underwent a tracheostomy and wide excision of the mass at the same time. A second diagnosis of hemangioma was also made by this center. The tracheostomy was closed successfully 21 days later.

**Figure 3:**
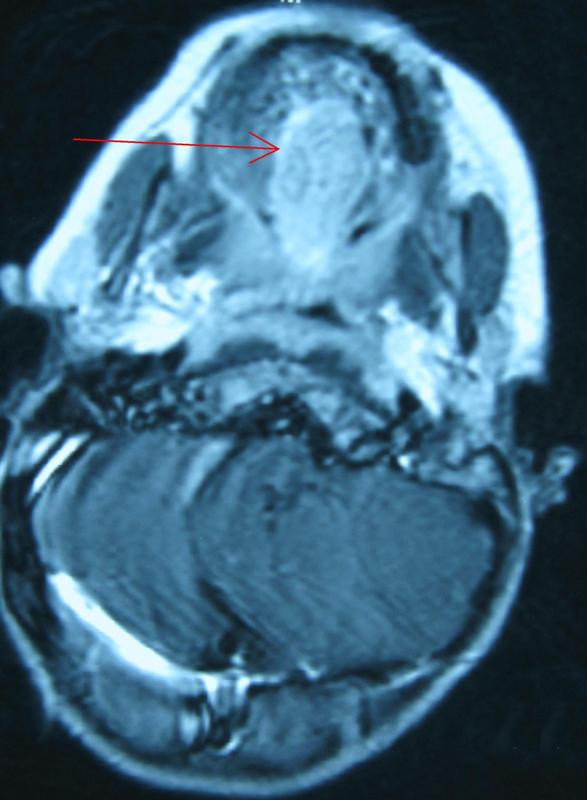
**Microscopic view showing nests of large granular cells separated by fibrovascular stroma (hematoxylin-eosin stain, magnification ×100)**.

Carefully controlled biopsies were then performed and the subsequent histological examination showed ASPS of the tongue. Microscopically the tumor consisted of structured, irregular and rough clusters of cells (Figure 3). Most of the vascular channels were enclosed by thin connective tissue septa. The tumor cells were large and polygonal with small and rounded nuclei. The cytoplasm of the tumor cells contained periodic acid-Schiff (PAS)-positive diastase-resistant material that was partly rod-shaped. This aspect has been observed in 80% of cases in other studies, and is helpful in differentiating this tumor from a rhabdomyoma. Rhabdomyomas also present PAS-positive cells, but the intracytoplasmatic glycogen granules disappear after pre-treatment with diastase [2].

**Figure 4:**
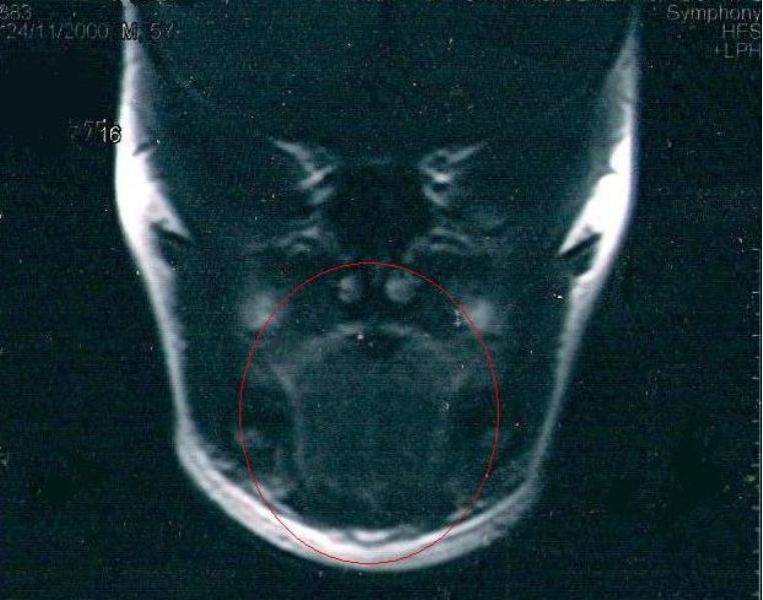
**One year after a complete excision of the sarcoma (red circle: post-operative area)**.

According to a series of cases reported, adjuvant therapy may not be necessary if the small primary lingual ASPS can be completely resected and the patient does not experience clinical recurrence or metastasis [3]. Therefore our patient was not treated with any post-operative chemotherapy as the removal of the tumor was considered to be an R0 ectomy (curative intent resection) and MRI, CT and scintigraphy of bones showed no local or distal metastasis. At three and a half years follow-up, our patient remained disease-free and asymptomatic (Figure 4).

## Discussion

Ôhe first case of ASPS was described by Christopherson *et al*. in 1952 [4,5]. Its clinical characteristics are slow growth and a high risk of mortality. The tumor occurs most commonly in the soft tissues of the extremities (27%) and rarely arises in the oral cavity, especially on the tongue (25%). Although there are a few reports of tumors occurring in the oral cavity, only 12 cases are reported in the English literature of ASPS presenting on the tongue in the first decade of life. Local recurrence of the tumor is rare, but metastasis to the lung, bones and brain may occur. The tumor occurs predominantly in females, with a female:male ratio of 2:1.

The pathogenesis of ASPS is not known and there is some controversy over this. One view is that it is of myogenic or neuroendocrine derivation. Although no theory has been specifically proven, the work of Mukai and colleagues [6] provides substantial support for the theory that it is of a myogenic origin. In some cases, a structural rearrangement of chromosome 17 involving band q25 has been reported [7].

The translocation (X; 17), producing an ASPL-TFE3 transcript fusion which is detected on tumoral cell walls, is specific to ASPS [8]. Patients with ASPS of the tongue experience dysphagia, dysphonia, or mild discomfort. Clinical examination often reveals pulsation with a thrill. Although there is usually no overlying ulceration or bleeding, our patient had significant hemorrhaging as the tumor extended through the overlying mucosa [9].

The typical histological characteristics of ASPS include a variable-size that is irregularly circumscribed, and division by thin fibrous connective tissue bands with delicate vessels delineating small pockets of variably-sized polygonal cells. The cells are of a uniform size and shape and contain granular, eosinophilic cytoplasm surrounding a vesicular nucleus that hosts a prominent nucleolus. The pseudoalveolar pattern characteristic of this tumor is seen when the alveolar cells undergo degeneration, lose their cohesiveness and become necrotic.

ASPS has a close clinical and imaging resemblance to common benign vascular tumors such as hemangiomas, which may lead to misdiagnosis and inadequate or delayed treatment. Vascular malformations may present at birth, whereas ASPS does not, but if it did it grows rapidly and occurs in an older age group. In addition to hemangiomas and vascular malformations, other less likely differential diagnoses include a hyperplastic lingual thyroid and dermoid cysts. These neoplasms are easily differentiated from ASPS in CT and MRI findings. ASPS can also be differentiated from other histologically similar malignant neoplasms in its cytological uniformity, lack of nuclear atypia, and paucity of mitotic figures. Most ASPSs also contain PAS-positive, diastase-resistant cytoplasmic inclusions that are thought to consist of actin. The differential diagnosis of ASPS includes granular cell tumor, paraganglioma, metastatic renal cell carcinoma, malignant melanoma, alveolar rhabdomyosarcoma and ectopic lingual thyroid [10].

The treatment of choice for ASPS of the tongue is generally surgical excision with sufficient margins. Surgery with chemotherapy or radiotherapy is useful in some patients. Treatment has been difficult to evaluate because of the small number of cases seen, but it seems that although the ectomy of the primary tumor is often successful, treatment of metastatic tumors is problematic [11]. For surgical excision, Marker *et al*. [12] recommend that a tumor-free zone of 1 to 1.5 cm should be maintained around the tumor.

Another approach to treatment is the use of IFN alpha-2b as a monotherapy. There is some evidence that high doses of IFN may induce an impressive tumor response in ASPS with pulmonary metastases that have no response to chemotherapy. However, this needs further research [13]. The prognosis for ASPS is poor overall, with five-year and 20-year survival rates of only 59% and 15%, respectively, being reported. Local recurrence and distant metastasis are primarily responsible for the poor prognosis of this neoplasm [14]. By contrast, distant metastases from oral cavity carcinomas vary over a broad interval (8-17%) and also depend on the stage of the disease [15]. Primary and metastatic tumors are generally treated by surgery, chemo-hormone therapy and immunotherapy [16]. The outcomes also depend on prognostic factors such as a patient's age, tumor size and the presence of metastasis at the time of diagnosis. In contrast to ASPS in other parts of the body, lingual ASPS have a rather good prognosis [7], particularly in young children.

## Conclusion

ASPS of the tongue is a rare tumor which occurs in infants and children, usually in the head and neck region. The orbital and lingual sites are predominantly affected. Treatment is primarily surgical, with limited roles for adjuvant chemotherapy, radiotherapy and perhaps IFN. CT and MRI can be used to make the correct diagnosis of ASPS and to help the surgeon to perform a wide surgical resection to reduce the risk of local recurrence. Additional study of the histological types of ASPS that occur in children, specifically in the head and neck region, is suggested to determine whether differences in patient outcome and treatment exist between the subtypes.

## Consent

Written informed consent was obtained from the patient's next-of-kin for publication of this case report and any accompanying images. A copy of the written consent is available for review by the Editor-in-Chief of this journal.

## Competing interests

The authors declare that they have no competing interests.

## Authors' contributions

GN analyzed and interpreted the patient data regarding the disease. PC performed the data analysis and was a major contributor in writing the manuscript. IP, KK and GK performed the operations. All authors read and approved the final manuscript.
